# An Enhanced Photosensitive Sensor Based on ITO/MWCNTs@Polymer Composite@BiVO_4_ for Quercetin Detection

**DOI:** 10.3390/bios13070729

**Published:** 2023-07-13

**Authors:** İrem Sarikaya, Esra Kaleoğlu, Soner Çakar, Cengiz Soykan, Mahmut Özacar

**Affiliations:** 1 Department of Chemistry, Faculty of Science, Sakarya University, Serdivan 54050, Türkiye; 2 Department of Chemistry, Faculty of Science, Zonguldak Bülent Ecevit University, Zonguldak 67100, Türkiye; esrakaleoglu@gmail.com (E.K.); cakarsoner@gmail.com (S.Ç.); 3 Biomaterials, Energy, Photocatalysis, Enzyme Technology, Nano & Advanced Materials, Additive Manufacturing, Environmental Applications and Sustainability Research & Development Group (BIOENAMS R&D Group), Sakarya University, Serdivan 54050, Türkiye; 4 Department of Material Science & Nanotechnology, Faculty of Engineering, Uşak University, Uşak 64200, Türkiye; cengizsoykan@usak.edu.tr

**Keywords:** quercetin, hydrothermal synthesis, photosensitive sensors, polymer composite sensors

## Abstract

The fact that antioxidants scavenge free radicals in the human body and naturally treat many health problems that will occur in this way has increased the consumption of antioxidant-containing foods. However, consumption of artificially prepared antioxidants could cause cancer. Therefore, antioxidants from natural sources are preferred. Quercetin is an antioxidant present in natural samples. In this article, multi-walled carbon nanotubes (MWCNTs), a polymer composite (PC) consisting of a mixture of 15% (by mass) polystyrene (PST), 15% (by mass) polyacrylonitrile (PAN) and 70% (by mass) polyindole (PIN), and semiconducting BiVO_4_ were used to prepare electrodes, and then a photosensitive ITO/MWCNTs@PC@BiVO_4_-based sensor was fabricated for quercetin detection. Quercetin was analyzed via the photosensitive ITO/MWCNTs@PC@BiVO_4_ sensor in 0.1 M phosphate buffered saline (pH 7.4) solutions including various quercetin concentrations. The constructed quercetin sensor displayed a wide linear response between 10 and 200 μM and a limit of detection of 0.133 μM. The developed photosensitive ITO/MWCNTs@PC@BiVO4 demonstrated a high sensitivity (442 µA mM^−1^ cm^−2^), good reproducibility (relative standard deviation 3.6%), high selectivity and long-term stability (>49 days) towards quercetin sensing. The photoelectrochemical sensor was then applied to detection of quercetin in black tea as a real-life sample. Our study could lead to the development of novel photosensitive PC polyphenol sensors.

## 1. Introduction

Flavonoids, found in the fruits, leaves, bark and roots of various higher plants, as well as in vegetables, grains, spices, flowers and tea, etc., are plant polyphenols [1]. In literature, some benefits of flavonoids include antifungal, anticancer, anti-inflammatory and antiviral features [2]. Flavanols is the largest subgroup of flavonoids. As a member of the flavanol family, quercetin (3,3′,4′,5,7-pentahydroxylflavone) is found abundantly in vegetables such as onions, garlic and scallions, as well as cocoa, green tea, and tea [3]. Quercetin is a phenolic compound ingested in the daily diet and has potential benefits for human health. Quercetin was demonstrated to offer benefits in some enzyme systems and the steps of biological processes of various diseases such as cardiovascular diseases, inflammation etc. [4].

Techniques including high-performance liquid chromatography (HPLC) [5,6], HPLC-mass spectrometry/mass spectrometry (MS/MS) [7,8], fluorescent methods [9,10,11], colorimetric techniques [12,13], chemiluminescent detection [14,15], biosensors [16,17], electrochemical- [18,19,20] and photoelectrochemical- [21,22] based sensors have been frequently used for quercetin analysis to date. Each of these techniques exhibit both superiorities and deficiencies. In recent years, the use of biosensors and sensors in quercetin analysis has increased due to the disadvantages of other techniques, such as the high cost of instrumental techniques (HPLC and HPLC–MS/MS), the difficulties in the application of fluorescent and chemiluminescent techniques, and low detection limits of colorimetric techniques at ~0.0033–0.0910 mM [12,13]. Electrochemical enzyme-based sensors have been applied to a wide field of study. However, these sensors have demonstrated disadvantages such as low stability, high cost, inability to operate below pH 2 and above pH 8, and decomposition at temperatures above 40 °C [23]. For this reason, scientists have been working intensively on non-enzymatic sensors in recent years. Although electrochemical techniques show features such as low detection limits (µM or nM level), photo-excited electrochemical techniques have attracted great attention of researchers for many years. Although photoelectrochemical techniques are a branch of electrochemistry and seem to be part of solar cells, their analytical applications also exist. Photoelectrochemical sensors are very sensitive due to the separation of photon excitation and detection [24]. In photoelectrochemical sensors, nano-conductive metal oxides are used as an effective photocatalyst due to their stability of about 400 s, selectivity, high sensitivity of 0.011 μA μM^−1^ cm^−2^, and low cost [23].

In many applications, polymeric materials that are chemically or molecularly inhomogeneous have been used in multi-component systems. Polymer nanocomposites have a wide range of new fillers that enhances the function and utility of polymers, while protecting the manufacturing and processing innate flexibility of thermosets, plastics, and resins. In particular polymer nanocomposites have been successful in terms of overcoming traditionally antagonistic combinations of features. Since the first reports in the early 1990s, [25,26,27,28,29] the term “polymer nanocomposite” has evolved to mean a multi-component system in which at least one dimension of the minor component is less than 100 nm and the major component is a polymer or a mixture thereof. Within the material class, conductive polymers have several characteristics including easy synthesis, macromolecular character, electrical handling properties, and being environmentally compatible. The design, synthesis, and modification of polymeric composites are becoming more and more important day by day, both in research and in the industrial area due to requests for applications in rechargeable batteries, sensors, electrochromic tools, anti-corrosion primer layers, and scaffolds for tissue engineering [30,31,32]. Polystyrene (PST) and polyacrylonitrile (PAN) are thermoplastic material compounds produced by homopolymerization of styrene (ST) and acrylonitrile (AN) that have the most important commercial applications in big requests due to their superior thermoplasticity, mechanical properties, dimensional stability, chemical and thermal stability, high resistance to alkalis and acids, craze resistance, stress resistance, easy processability, and a stress–strain behavior, which is comparable to the linear elastic/perfect-plastic materials [33,34]. PST and PAN are important industrial plastics and interesting candidates as components in the synthesis of conductive composites. Polyindole (PIN) is particularly promising for industrial applications because of its easy synthesis, high redox activity and higher conductivity. Unlike other conductive polymers, PIN may be synthesized by chemical oxidation of indole in different organic solvents [35]. In this study, a structure containing 15% polystyrene (PST), 15% polyacrylonitrile (PAN) and 70% polyindole (PIN) was used as a conductive polymer composite. Polystyrene (PST) and polyacrylonitrile (PAN) were chosen as an insulating matrix. PIN is used as a conducting matrix to facilitate electron transfer in the ternary composites due to its high conductivity [36]. In addition, the polymeric structures are frequently used as bonding agents between semiconductor materials due to their long-chain nature.

In the literature, there are studies on electrochemical and photoelectrochemical methods for the analysis of quercetin, and these studies are generally related to the use of sensors based on noble metal or metal oxide structures on a carbon support material. Therefore, in this study, a photoelectrochemical quercetin sensor was evolved by coating a conductive polymer composite (PST/PAN/PIN mixture denoted as PC) and photosensitive BiVO_4_ semiconductor on a new-generation, multi-walled carbon nanotube (MWCNT) support. In recent years, studies with carbon-based materials have received great attention due to their unique ability to detect small molecules such as glucose, dopamine, quercetin, urea and H_2_O_2_ [37,38,39]. In addition, MWCNTs are used both as a catalyst and as a support material. In photosensitive sensors, light-sensitive materials are generally used to increase the photocurrent. Among these materials, BiVO_4_ is used as a photosensitive material in the visible region due to its wide band gap, good stability and low toxicity. Light-sensitive sensor studies using BiVO_4_ structures in the literature have gained importance in recent years [40,41]. The materials used in this innovative non-enzymatic sensor structure were chosen for several reasons: the excellent conductivity (49.49 S/cm) and high electron mobility (~50,000 cm^2^ V^−1^ s^−1^) characteristics of MWCNTs; the PC’s ability to facilitate electron transmission and linker properties; and BiVO_4_’s good light-harvesting features. BiVO_4_ has a low band gap energy (∼2.3–2.4 eV) and the capacity to absorb most of the solar spectrum [42]. The MWCNTs’ structure used in this study is generally used in the analysis of compounds such as quercetin for electrochemical detection. In addition, the BiVO_4_ light-sensitive semiconductor structure used in this study has been used in applications such as photocatalysis and solar-driven water splitting in the literature. Due to its light-sensitive nature, BiVO_4_ has been used in sensor applications in the analysis of various organic structures before, but as far as we know, there is no study on quercetin analysis. In addition, it was observed in this study that a good electron transfer was achieved with the developed triple composite polymer structure and that there was a good connection between MWCNTs and BiVO_4_. For these reasons, our study, which consists of well-known materials in the literature, has a unique value due to its quercetin sensor, innovative composite polymeric structure, photosensitivity and explanation of the quercetin mechanism. MWCNTs, PC and BiVO_4_ were correspondingly coated on an indium tin oxide (ITO) glass substrate by a drop casting method. The synthesized nanostructures and fabricated sensor were characterized via X-ray diffraction (XRD), Fourier transform infrared (FTIR) spectroscopy, field emission scanning electron microscopy (FE-SEM), elemental mapping, energy dispersive spectroscopy (EDS), Mott–Schottky analysis, UV-Vis absorbance (UV-Vis) and diffused reflectance spectroscopy (DRS) techniques. Electrochemical impedance (Nyquist and Bode), amperometric (i-t) and linear sweep voltammetry (LSV) techniques were used for the electrochemical characterization of the quercetin sensor produced by correspondingly coating MWCNTs, PC and BiVO_4_ on an ITO substrate. The ITO/MWCNTs@PC@BiVO_4_ photosensitive sensors showed good selectivity, stability, low limit of detection (LOD) and broad linear response range for detecting quercetin. In addition, quercetin was analyzed in Turkish traditional tea (ÇAY) using the developed photoelectrochemical sensor.

## 2. Materials and Methods

### 2.1. Reagents

After removal of the hydroquinone inhibitor in styrene (ST-Sigma-Aldrich, Burlington, MA, USA) and in acrylonitrile (AN-Sigma-Aldrich, Burlington, MA, USA) monomers with a dilute potassium hydroxide solution, these monomers were used in the preparation of polystyrene (PST) and polyacrylonitrile (PAN). Indole (purity ≥ 99%) monomer was obtained from Merck Company, Germany. Benzoyl peroxide (C_14_H_10_O_4_; BPO) was utilized to initiate free radical polymerization of styrene and acrylonitrile monomers after being dissolved in chloroform and recrystallized via precipitation in methanol. Nitrogen was used to provide an inert atmosphere during the polymerization process. To remove impurities from the polymers, 1,4-dioxane was used for solubilization and ethanol for reprecipitation. Chloroform, 1,4-dioxane and ethanol were acquired from Merck Company, Germany and utilized in experimental studies without any purification.

### 2.2. Synthesis of Homopolymers of Styrene and Acrylonitrile

In our work, 8 g of styrene monomer and 8 g of acrylonitrile monomer were transferred into two separate polymerization tubes. Then, 1,4-dioxane was used to dissolve the monomers. Afterwards, 1% by mass (0.08 g) of the monomer C_14_H_10_O_4_ was used as the polymerization precursor at 70 °C. The polymerization medium was rendered inert through the use of nitrogen gas. The synthesized homopolymers were purified by dissolving in 1,4-dioxane three times and precipitating with ethyl alcohol to remove impurities. The obtained homopolymers were firstly dried in ambient conditions and then at 40 °C in a vacuum oven and kept in bottles for composite synthesis.

### 2.3. Synthesis of Ternary PST/PAN/PIN Conductive Polymer Composite

**Figure 1:**
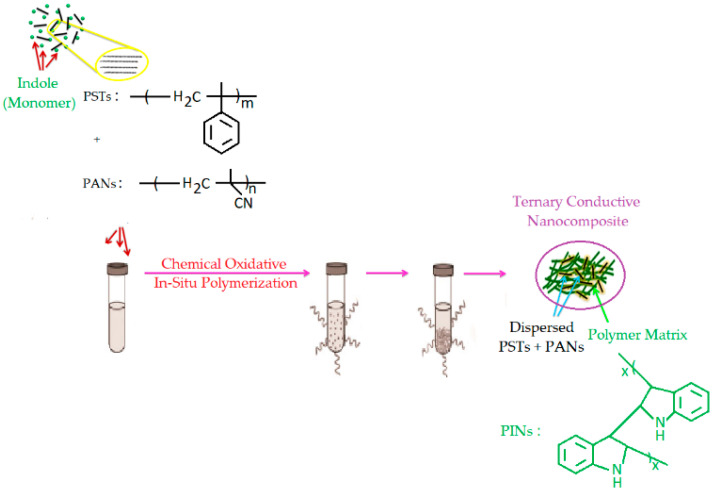
Schematic diagram of preparation of PST/PAN/PIN ternary conductive composite.

The reaction diagram of the synthesized ternary conductive PC (PST/PAN/PIN) is shown in Figure 1. The PST/PAN/PIN ternary PC was synthesized via the chemical oxidative polymerization method at 25 °C using the oxidant FeCl_3_. An oxidant-to-monomer mole ratio of 2:1 was used. In the first stage, 7.25 g of FeCl_3_ was dissolved in 30 mL chloroform in the reaction tube. In the next stage, 1.0 g of PST and 1.0 g of PAN were dissolved in 20 mL chloroform so that the total polymer mass was 2 g, and these were added to the oxidant solution mixed in the reaction tube. After the mixture in the reaction tube was stirred for 15 min, 2.62 g of indole monomer dissolved in chloroform was added dropwise to this mixture using a dropping funnel under N_2_ atmosphere at a fixed 25 °C reaction temperature. The product was separated by filtration from the reaction mixture, which was stirred for 30 h, and washed first with chloroform and then with hot distilled water. In the last stage, the obtained product was dried for 24 h at 45 °C in a vacuum oven. 

### 2.4. Synthesis of BiVO_4_ Nanoparticles 

In this work, 2.425 g Bi(NO_3_)_3_∙5H_2_O and 0.585 g NH_4_VO_3_ were dissolved in 40 mL ultrapure water and mixed for 30 min on a magnetic stirrer. Then, 1 M NaOH was added to bring this solution into the pH range of 10–12. This mixture was transferred into a Teflon-based hydrothermal vessel, heated to 180 °C and kept there for 18 h. The resulting product was washed several times with distilled water and methanol to eliminate impurities, and then dried at 80 °C in an oven overnight. The obtained BiVO_4_ nanoparticles (NPs) were characterized via XRD, FTIR and FE-SEM techniques.

### 2.5. Preparation of Electrodes 

The photosensitive sensor was prepared by coating on ITO substrates. Firstly, the ITO electrode was cleaned in ultrapure water, acetone, ethanol/water (1:1) mixture and 0.1 M NaOH solution by sonication, respectively. Secondly, separately 2 mg/L BiVO_4_, 2 mg/L MWCNT and 2 mg/L PC were dispersed in methanol and vigorously stirred for 30 min for homogenization. Thirdly, 4 μL MWCNTs, 4 μL PC and 4 μL BiVO_4_ were correspondingly introduced to the ITO substrate, then dried under ambient conditions and kept in the dark. The constructed ITO/MWCNT@PC@BiVO_4_ electrode was used as a photosensitive sensor in the analysis of quercetin.

### 2.6. Characterization of Synthesized Materials and Prepared Photosensitive Sensors

The photocurrent measurements were performed under 150 W halogen lamp (tungsten halogen double-ended (J-type)) irradiation. XRD analysis was achieved by PANalytical–Empyrean X-ray diffractometer. FTIR analysis was performed by a Perkin Elmer Frontier FTIR spectrometry with ATR equipment. Thermogravimetric analysis (TGA) was performed by a TGA/differential thermal analysis (DTA) device (Hitachi 7000). The Mott–Schottky measurements of prepared ITO-coated samples were analyzed using 0.1 M LiCO_4_ solution on a potentiostat/galvanostat/zero resistance ammeter (ZRA) (Gamry Interface 1000, Gamry Instruments, Warminster, PA, USA). The UV-Vis absorption and DRS spectroscopic measurements were obtained using a Schimadzu UV-2600 spectrometer. FE-SEM analysis was performed using a Philips XL30 SFEG microscope equipped with an EDS (INCA X-Max 80). All electrochemical analyses (linear sweep voltammetry (LSV), amperometry (i-t) and cyclic voltammetry (CV)) were conducted using a Gamry potentiostat/galvanostat/zero resistance ammeter (ZRA) in the presence of 0.1 M phosphate buffered saline (PBS, pH = 7.4) under an N_2_ atmosphere. Electrochemical impedance spectroscopy (EIS) analyses, and the Nyquist and Bode plots, were performed under dark and light exposure at short-circuit bias superimposed by an AC voltage of 10 mV peak-to-peak amplitude over a 0.1–10^6^ Hz frequency range.

## 3. Results

### 3.1. FTIR Spectra Analysis of PST, PAN, and Ternary PST/PAN/PIN Composite

**Figure 2:**
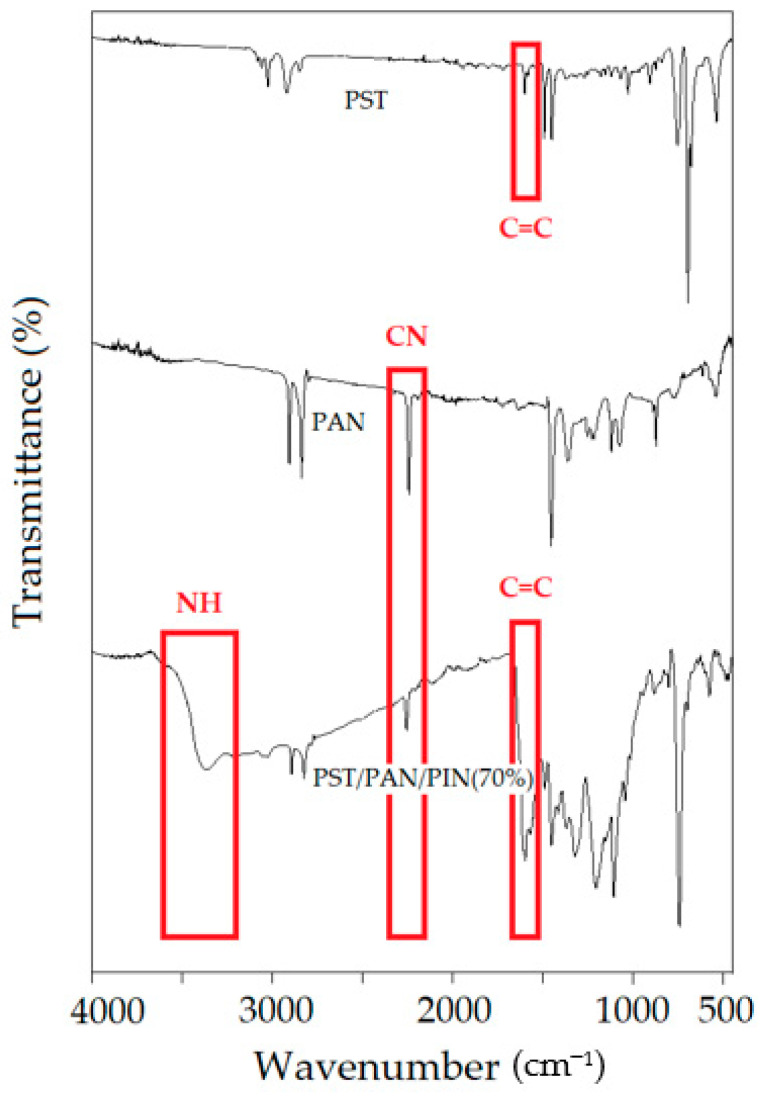
FTIR spectra of PST, PAN and PST/PAN/PIN.

In Figure 2, the FTIR spectra of the PST, PAN and ternary PST/PAN/PIN composite (PC) are presented. In the PST FTIR spectrum, characteristic absorption bands were observed at 3030–2900 and 1600–1450 cm^−1^ attributable to the C−H stretching vibration and the C=C stretching vibration of the benzene ring, respectively, and these have been previously reported for PST [43]. The bands at 2252 cm^−1^ and 1616 cm^−1^ were recorded as the main characteristic peaks corresponding to C≡N and C–C bonds in the FTIR spectrum of PAN. For the PC, the existence of peaks at 1444 cm^−1^ is attributed to C=C, 2252 cm^−1^ is related to C≡N, and 3390 cm^−1^ belongs to N−H stretching in the PIN unit.

### 3.2. Thermogravimetric Analysis of PST, PAN, PIN and Ternary PST/PAN/PIN Composite

**Figure 3:**
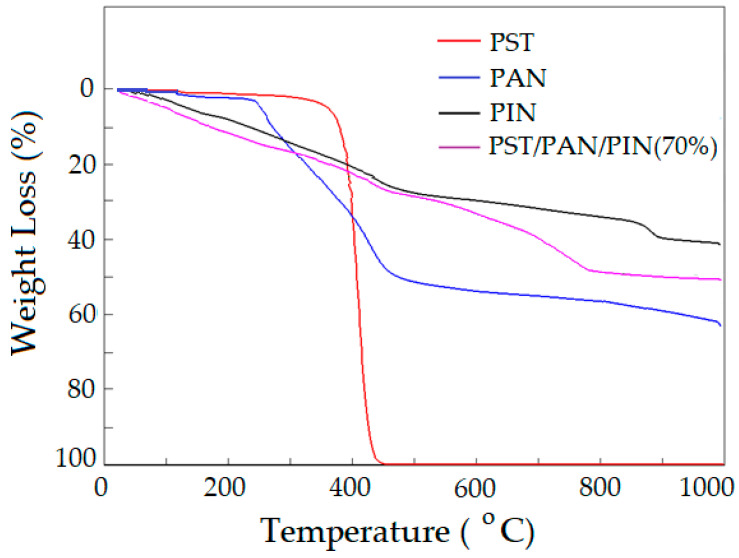
TGA thermograms for PST, PAN, PIN and PST/PAN/PIN.

The thermogram of the ternary PST/PAN/PIN composite produced via the chemical oxidative in situ polymerization method, analyzed from room temperature to 1000 °C at a heating rate of 10 °C/min, is given in Figure 3 together with the thermograms of PST, PAN and PIN. From the decomposition curve of the PST, it appears that the PST is stable above 270 °C. The PST decomposed up to 400 °C and the whole sample transformed into CO or CO_2_ gas, completed at 430 °C. This is a typical result of PST reported in the literature [44]. The TGA thermogram of the PAN illustrates two main decomposition steps. In the initial step, the degradation of PAN is in the range between 234 °C and 305 °C and in the second step is in the range of 305 °C and 420 °C. The total weight loss arises from the disappearance of volatile components such as CO, CO_2_ or NO_x_ and the formation of cyclic compounds between the neighboring –CN groups [45]. Due to its heterocyclic ring structure, indole appears to be thermally stable up to 1000 °C. The thermal stability of the PC shows an intermediate behavior compared to individual PST, PAN, and PIN polymers. This situation is attributable to the new formations developed between molecules in the ternary composite structure. As seen from Figure 3, the thermal stability of the composite formed—due to the covalent bonds, pi-pi interactions and strong hydrogen bonds it contains—has increased compared to the individual polymers.

### 3.3. Composite Structure Characterizations 

**Figure 4:**
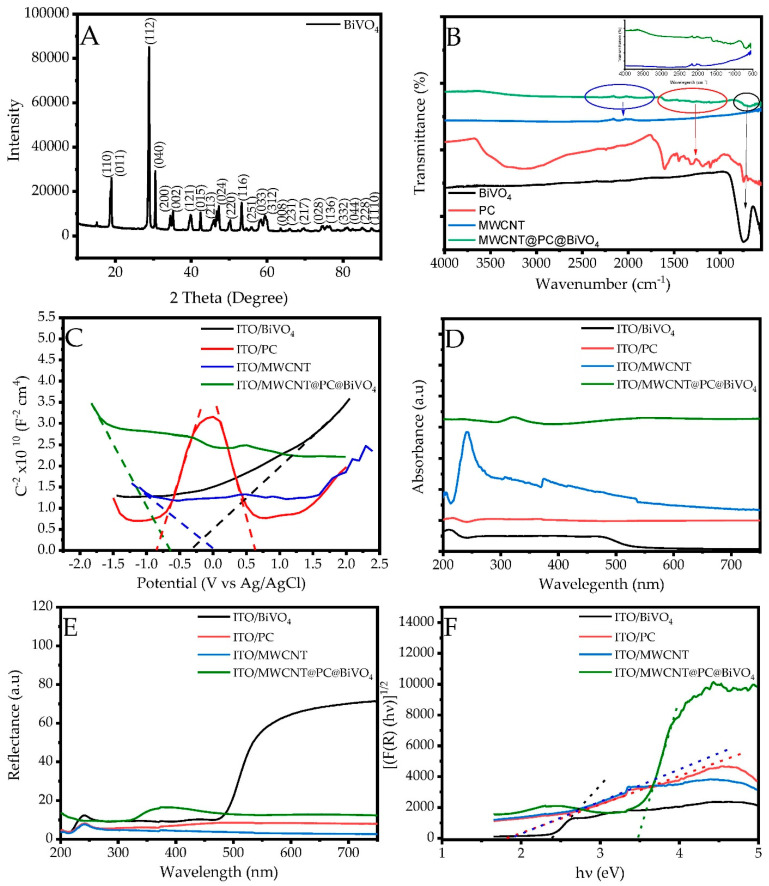
(**A**) XRD spectrum of BiVO_4_, (**B**) FTIR spectra of BiVO_4_, PC, MWCNT and MWCNT@PC@BiVO_4_ electrodes, (**C**) Mott-Schottky, (**D**) UV-Vis absorption spectra, (**E**) DRS spectra and (**F**) Kubelka–Munk graphs of prepared ITO/BiVO_4_, ITO/PC, ITO/MWCNT and ITO/MWCNT@PC@BiVO_4_ electrodes.

The XRD spectrum of BiVO_4_ NPs synthesized via the hydrothermal method is displayed in Figure 4A. The synthesized BiVO_4_ NPs have a monoclinic crystal structure and the XRD pattern is in agreement with that reported in the Inorganic Crystal Structure Database (ICSD) code 98-010-0605. The unit cell parameters of synthesized BiVO_4_ NPs, a, b, c, volume (V), space group are 7.234 Å, 11,702 Å, 5.093 Å, 310.01 Å3, I 12/c1, respectively. In addition, based on our previous work [46], the crystal size and microstrain of the BiVO_4_ NPs are estimated to be 26.16 nm and 0.526%, respectively. From the XRD spectrum, it is seen that BiVO_4_ does not contain impurities, and all the peaks are in a good harmony with the BiVO_4_ structure and hkl indices.

The FTIR spectra of the composite electrode consisting of MWCNTs, PC and BiVO_4_ and a mixture of these samples are given in Figure 4B. The peak seen at 747 cm^−1^ in the FTIR spectrum belongs to the BiVO_4_ NPs is due to the V-O bond [47]. In the FTIR spectrum of the PC, the large peak seen at 3500–3000 cm^−1^ is due to the –OH bond. In addition, the peaks seen in the 1700–700 cm^−1^ region belong to aromatic C-C, C-O, O-H bonds. The structures corresponding to the peaks seen at the wavenumbers specified in the FTIR spectrum of PC are as follows; aromatic C-C/C=C stretching mode (1607), aromatic CH_2_ ring vibration (1487), aromatic C-O-H in plane blending (1446), -OH and C-H stretching (1368), C-O stretching (1317), C-OH stretching and C-H in plane deformation (1107), O-CH_3_ and C-OH stretching (1063), C-O stretching (1036, 1014), C-H out of plane bending (928), ring out of plane C-H bending (802), C-H out of plane bending vibration (746), -OH bending (693) [48,49]. The y-axis expanded FTIR spectra of MWCNTs and the ternary composite electrode material are presented in the inset of Figure 4B. As seen from the FTIR spectrum of MWCNTs, the peaks at 2157, 2021 and 1570 cm^−1^ is due to C=C [50]. As examined the FTIR spectrum of the ternary composite electrode fabricated with BiVO_4_, PC and MWCNTs, the large peak of BiVO_4_ structure at 750 cm^−1^, the peaks of the PC structure in the 1700–1000 cm^−1^ region, and the MWCNT C=C peak regions are clearly seen. 

**Figure 5:**
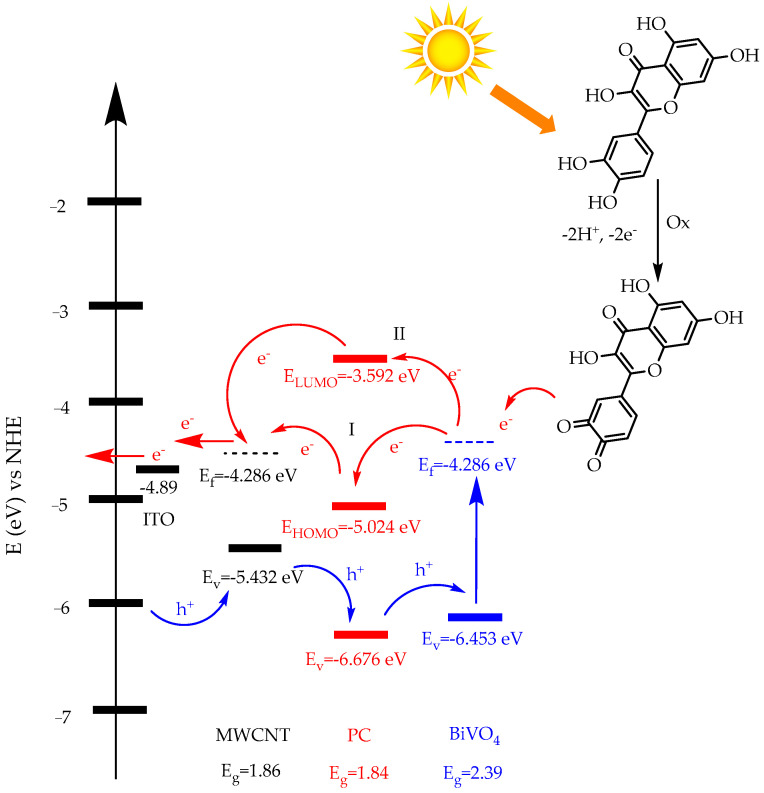
The energy band diagram of prepared non-enzymatic quercetin sensor based ITO/MWCNT@PC@BiVO_4_.

The Mott–Schottky curves of the ITO/BiVO_4_, ITO/MWCNT, ITO/PC, and ITO/MWCNT@PC@BiVO_4_ composite electrode are shown in Figure 4C. In Mott–Schottky measurements, the flat band potential of the prepared electrode has been determined from the linear slope of the 1/C^2^ against V plot by employing the following equation;

(1) $$\frac{1}{C^{2}}=\left\lceil \frac{2}{q\;N_{D}\;K\;\varepsilon_{0}A^{2}}\right\rceil \left(V-V_{fb}-\frac{k_{B}T}{e}\right)$$

where, *C^2^* is interfacial capacitance, *A* is an interfacial area, *N_D_* is the number of donors, *V* is the applied voltage, *k_B_* is Boltzmann’s constant, T is the absolute temperature and e is the electronic charge. The *V_fb_* can be determined from the intercept of the *V* axis. The flat band potentials (*E_f_*) for ITO/BiVO_4_, ITO/MWCNT and ITO/MWCNT@PC@BiVO_4_ were correspondingly estimated to be −0.114, 0.213, −0.447 V vs NHE. As can be seen from the Mott–Schottky spectrum of the ITO/PC structure, there are two different flat band potential values. The points where the curves in the dashed lines cut the x-axis (red dashed lines) in Figure 4C correspond to the flat band potentials of the polymer structure. With the help of the Mott–Schottky equation, the graph of 1/C^2^ against V shows two flat band potentials, n and p type, in the PC structure. This shows that the conductive polymer has p-n junction properties [51]. In terms of providing the interconnection between n type BiVO_4_ and p type MWCNT, the p-n connection feature of the conductive polymer structure makes a positive contribution to the sensor properties of the composite structure by reducing the resistance and increasing the conductivity, as it facilitates electron transmission. The p type and n type E_f_ values of conductive polymer were determined as 0.823 and −0.651 V against NHE, respectively. Additionally, the UV-Vis absorption spectra and DRS of the ITO/BiVO_4_, ITO/PC and ITO/MWCNT and ITO/MWCNT@PC@BiVO_4_ electrodes are shown in Figure 4D and Figure 5E, respectively. As seen in the UV-Vis absorbance spectra, the prepared structures absorb light in the visible region. In addition, it is seen that MWCNTs show strong absorbance in the UV region, whereas the absorbance of the BiVO_4_ is potent in the Vis region. Thus, the electrode components show a synergistic effect in the prepared composite electrode, indicating that both the UV region and the Vis region of the light will be strongly absorbed and the current properties of the sensor will be increased under light. In addition, DRS analyses of the prepared structures were performed and band gap values were calculated with the help of the Kubelka–Munk function. The Kubelka–Munk function was used to identify the optical band gap (E_g_) as follows [52,53];

(2) $$F\;\left(R\right)=\frac{{\left(1-R\right)}^{2}}{2R}=\frac{K}{s}$$

where, *F (R), R, K* and *s* correspondingly represents the Kubelka–Munk function, reflectance, absorption and scattering coefficient. The Kubelka–Munk plots ([F(R)hν]^2^ versus photon energy (hν)) calculated from the reflectance spectra in Figure 4E are shown in Figure 4F. E_g_ was estimated by the abscissa intercept of the tangent to the rising segment of the plot. The E_g_ values of ITO/BiVO_4_, ITO/MWCNT, ITO/PC, and ITO/MWCNT@PC@BiVO_4_ electrodes are correspondingly 2.39, 1.86, 1.84 and 3.47 eV, as illustrated in Figure 4F. Unexpectedly, the band gap value of the composite electrode was higher than the single electrodes. The main reason for this is thought to be due to the fact that n type BiVO_4_, p type MWCNT and p-n junction PC electrode are conjugated in the form of p-n-p-n. Band gap values calculated for single electrodes are in good agreement with literature data [54,55]. Valence band potential (E_v_) values of electrodes can be estimated by subtracting the E_f_ values obtained from Mott–Schottky curves from the E_g_ values calculated via the Kubelka–Munk function. The calculated E_v_ values for ITO/BiVO_4_, ITO/MWCNT, ITO/PC, and ITO/MWCNT@PC@BiVO_4_ electrodes are correspondingly −6.453, −5.432, −6.676 and −7.423 eV. In particular, the low E_v_ value of the prepared composite electrode led to the low energy value.

The energy diagram of the movements of electron and hole pairs in the developed sensor is shown in detail in Figure 5. Quercetin exposed to sunlight turns into quercetin-on quinone by donating electrons, and the released electrons flow over the flat band of BiVO_4_ semiconductor and then through PC and MWCNTs. In the BiVO_4_ structure, released electrons move through two different pathways. The main reason for this is that the synthesized PC structure behaves like a p-n junction. Electrons released in path I pass to the HOMO energy level of the PC, then pass to the flat band of the MWCNTs, and then flow through the external circuit and form an electrochemical signal. In pathway II, the oscillating electrons in the conduction band of BiVO_4_ pass into the LUMO energy band of the PC and then pass into the flat band of the MWCNTs, producing an electrochemical response. Holes (h^+^) formed due to electron movement move from the MWCNTs valence band to the PC valence band and then to the BiVO_4_ valence band. In particular, the synthesized PCs acting as a p-n junction allows for increased electron movement and improved sensor properties. In addition, it is clearly seen that the used BiVO_4_ NPs are light-sensitive due to their band properties [40,41,42]. The scheme shows that this new generation ITO/MWCNTs@PC@BiVO_4_ sensor developed should be effective in the quercetin analysis.

**Figure 6:**
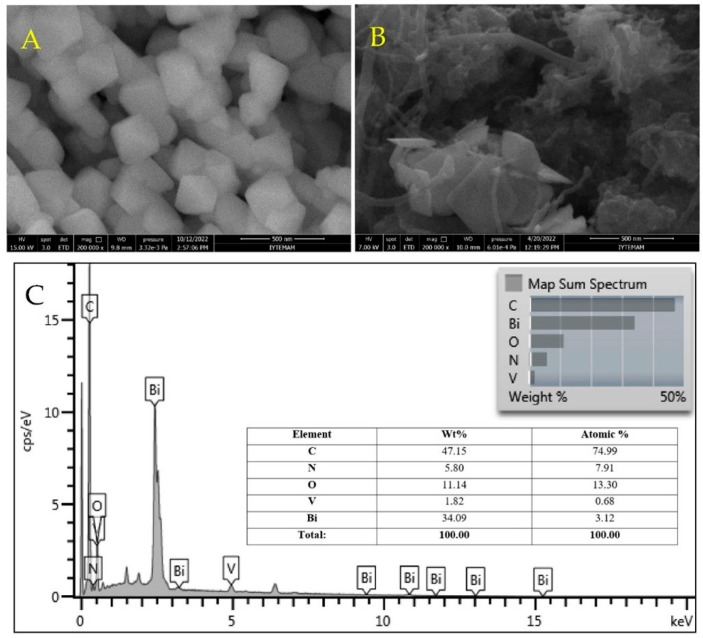
FE-SEM images of (**A**) BiVO_4_ and (**B**) MWCNT@PC@BiVO_4_ composites and (**C**) EDX spectrum of MWCNT@PC@BiVO_4_ composites.

**Figure 7:**
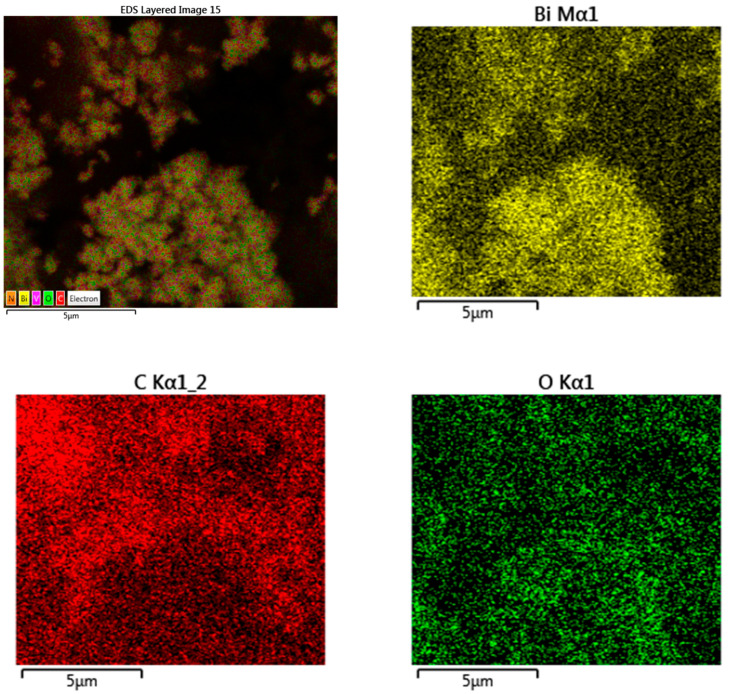

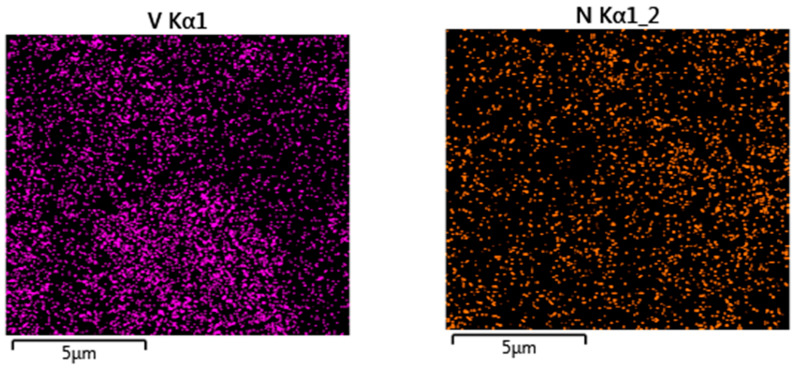
Elemental mapping images of prepared MWCNT@PC@BiVO_4_ composites.

The field-emission scanning electron micrograph of synthesized BiVO_4_ NPs is shown in Figure 6A. BiVO_4_ NPs with a tetrahedral structure have a diameter range of 50–100 nm. In addition, the field-emission scanning electron micrographs of the electrode composite (MWCNTs@PC@BiVO_4_) are displayed in Figure 6B. The fiber-like structure that can be seen in the micrograph in Figure 6B belongs to MWCNTs, tetragonal structures to BiVO_4_ NPs, and small spherical structures to the polymer. The average particle size of the PC is 10–50 nm, and the MWCNT structure is 1–3 nm in diameter and 300–500 nm in length. In addition, the EDX spectrum of the electrode composite (Figure 6C) shows the elements that make up the composite and their combinations. The EDX spectrum also shows that the prepared electrode composite does not contain any impurities. In addition, the atomic and mass ratios of the atoms that make up the composite are shown in the inset of Figure 6C. Considering the atomic ratios, Bi and V elements originate from the BiVO_4_ NPs structure, and N from the PC. The C and O elements originate from the MWCNTs, PC and BiVO_4_ structure. Elemental mapping images of the prepared MWCNTs@PC@BiVO_4_ composite electrode are displayed in Figure 7. As seen in the elemental mapping images, each element is uniformly distributed in the composite electrode. It is observed that the N atoms, where the Bi and V elements are distributed together, are also less dense elementally than the other compounds and are distributed on the general composite. 

### 3.4. Light Sensitive Sensor Applications

**Figure 8:**
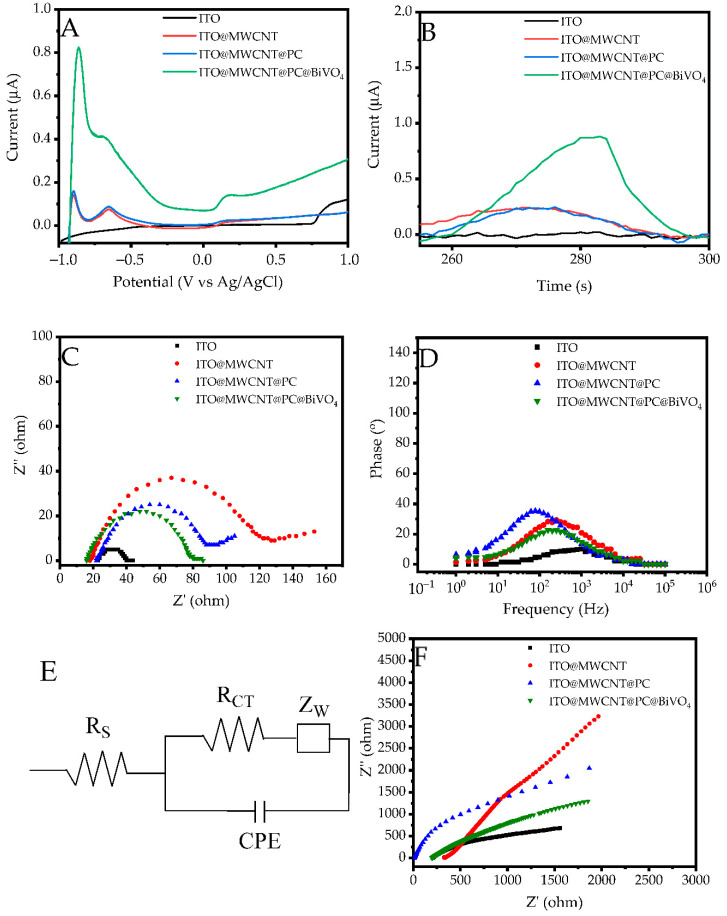
(**A**) Photocurrent vs. potential (LSV) curves, (**B**) time dependent photocurrent (i-t), (**C**) Nyquist plot, (**D**) Bode plot, (**E**) equivalent circuit model and (**F**) dark mode Nyquist plot in 100 µM quercetin solution on light of fabricated sensors.

A photosensitive quercetin sensor was constructed using MWCNT, conductive PC and BiVO_4_ NPs. In the first stage, the photosensitivity of these materials was determined by taking the LSV curves of the coatings on ITO glass. As depicted in Figure 8A, linear sweep voltammograms of 100 µM quercetin show various electrode composites on an ITO glass in 0.1 M PBS as a supporting electrolyte. The current increased sharply from 0.15 µA to 0.8 µA after BiVO_4_ coating in composite systems due to the stimulation of the quercetin structure at −0.85 V, thus also increasing the quercetin-sensing capacity of the electrode. The visible light-mediated activity of the composite electrode can be attributed to the photoactive nature of BiVO_4_. In contrast to BiVO_4_, a very weak current increase (~5%) was observed for MWCNT and conductive PC electrodes. In addition, it was determined as −0.85 V operating voltage, which gives the highest current value (0.82 µA) from the LSV curves. After the operating voltage was determined, the photocurrent response (i-t) curves of the same substances were taken (Figure 8B), and it was seen that the current in BiVO_4_ NPs increases significantly from 0.27 to 0.82 µA depending on the light at the voltage determined as −0.85 V. Since conductive polymers are known to accelerate electron conduction in their located systems [10], in this study, PC was used to improve electron transfer between BiVO_4_ and MWCNT structures due to its high electronic mobility (~1000 cm^2^ V^−1^ s^−1^). In addition, the PC has been used to help MWCNTs and BiVO_4_ NPs bond together, thereby increasing the durability of BiVO_4_ NPs at the electrode surface. Dark and light electrochemical impedance curves and conductivity properties of the samples coated on the ITO glass were analyzed. Nyquist plots of 100 µM quercetin at the prepared electrode composite on ITO in 0.1 M PBS as supporting electrolyte at the light exposure are shown in Figure 8C. As examined the Nyquist curves, it is seen that the ITO electrode exhibits low resistance (17.44 Ω). R_S_ generally denotes the solution resistance and is estimated by the starting point x axis of the Nyquist plot; R_CT_, the charge transfer resistance of the redox species used in the experiment at the electrode/electrolyte interface, which is calculated by the semicircle resistance minus R_S_; C_μ_ the capacitive phase element (including the double-layer capacitance and electrode inhomogeneity) and is calculated from fitted curves of the Zview program [56,57]. The f_max_ is the maximum frequency point of prepared composites and is obtained from Bode plots. Calculated electrochemical impedance values are shown in Table 1. As the calculated electrochemical impedance values show, it is seen that while the R_CT_ resistance increases significantly from 17.44 to 104.84 Ω with the coating of MWCNT on ITO, the resistance decreases greatly to 66.13 Ω value with the coating of PC on ITO/MWCNT. Among the prepared electrodes, the ITO/MWCNT@PC@BiVO_4_ electrode had the lowest resistance value of 60.75 Ω under the light. This indicates that the conductivity behavior of the prepared composite structure increases with light. In addition, as observed in the Bode-type curves (Figure 8D), the ITO/MWCNT@PC@BiVO_4_ structure has the lowest f_max_ value. The lowest f_max_ value is used in the calculation of electron lifetime (τ_e_) passing through the sensor. While the electron lifetime is calculated with Equation (2), the diffusion time (τ_d_) value can be calculated with the help of Equation (3) by using the R_CT_ and C_µ_ values obtained from the Nyquist curves [57].

τ_e_ = 1/(2 π f_max_) (3)

τd = RCT × Cµ(4)

The τ_e_ and τ_d_ values calculated via Equations (2) and (3) are given in Table 1. Notably, the conductivity and electron mobility respectively increased by 20.03% and 1,45% after both MWCNTs and PC were secured on the electrode, compared to ITO/MWCNTs. Relative to ITO/MWCNT@PC, τ_e_ has increased by 282% after BiVO_4_ NPs were included on the electrode. As τ_e_ is a feature that represents the movement of the electron in the circuit along the circuit, it is a parameter that will aid in enhancing the conductivity of the sensor. Similarly, τ_d_ for ITO/MWCNT@PC@BiVO_4_ structure is the highest compared to all other electrodes. The main reason for this is every additional component added to the structure affects the cut-off time. When both τ_e_ and τ_d_ values are evaluated together, it is seen that they have an effect on increasing electrochemical conductivity under light. It can be said that especially BiVO_4_ NPs in the sensor structure increase electron movement and electron capture due to their photosensitive nature. The equivalent circuit model of the fabricated composite ITO/MWCNT@PC@BiVO_4_ electrode is shown in Figure 8E. This model was obtained by simulating the Zview program. In this model, R_S_, R_CT_, Z_W_ and C_μ_ represent the layer resistance, the ITO/electrode surface charge transfer resistance, the Wargburg impedance value of the ion diffusion, and the capacitive phase element values, respectively. Dark mode impedance results are depicted in Figure 8F. These curves also show similar properties with the impedance results obtained under light. It is seen that the resistance values are lower for the measurements taken under light, whereas the dark impedance measurement results show higher resistance values. Similarly, it is observed that the ITO/MWCNT@PC@BiVO_4_ electrode exhibits lower resistance in the dark Nyquist curve than the other electrodes. It should be noted that the resistance of the ITO electrode, which is not coated with any material, is approximately 55% lower than the ITO/MWCNTs@PC@BiVO_4_ electrode. 

**Figure 9:**
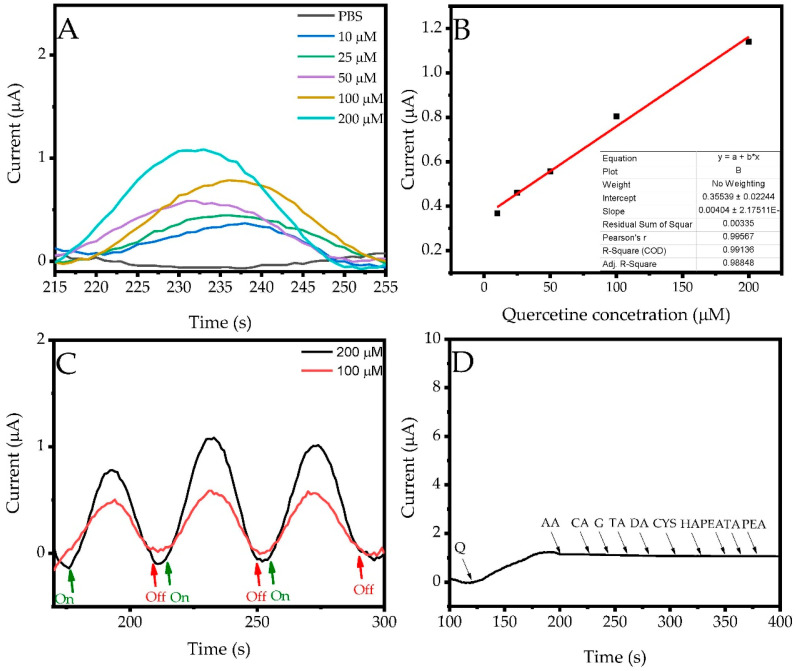
(**A**) Amperometric I-t curves, (**B**) calibration curves of different concentrations of quercetin, (**C**) on-off switching curves and (**D**) interference study.

Time-dependent photocurrent (i-t) curves at various quercetin concentrations are represented in Figure 9A. An increase in photocurrent was observed as the quercetin concentration increases. The resulting calibration graph is shown in Figure 9B. Quercetin is oxidized at the applied potential of −0.85 V to form oxidized quercetin (quercetin quinone). The electrons generated are transferred to the conduction band of BiVO_4_. Electrochemical signal is then generated by flowing electrons from the conduction band of BiVO_4_ through the conducting PC and MWCNT, respectively. The conduction mechanism of the fabricated sensor is given in Figure 5 (vide supra). As can be seen from the calibration curve, the fabricated sensor operates linearly in the concentration range of 5–200 µM. In addition, the LOD (evaluated based on a signal-to-noise ratio of 3), limit of quantification (LOQ) (based on a signal-to-noise ratio of 10), and sensitivity values were determined as 0.133 μM, 0.443 μM, and 442 µA mM^−1^ cm^−2^, respectively. These calculated values and their comparisons with some quercetin sensors in the literature are presented in Table 2. As can be seen from the 200 µM and 100 µM on-off switching curves (Figure 9C), it is observed that the peak intensity increases under light due to the light-sensitive structure of the fabricated sensor and decreases with the light off. In addition, it is seen that the synthesized composite structure also has instant sensitivity to light. Interference studies were carried out using various compounds such as ascorbic acid (AA), citric acid (CA), glucose (G), tartaric acid (TA), dopamine (DA), cysteine (CSY), histamine (HA), phenethylamine (PEA) and tyramine, containing –OH and –NH_2_ groups. They can coexist with quercetin in natural products and may interfere with it during quercetin detection. The selectivity of the fabricated sensor was investigated in solutions containing 100 µM quercetin in the existence of selected interference compounds and the results are shown in Figure 9D. The developed sensor operates at a high voltage value of −0.85 V, which was determined as the operating voltage in the previous section (Figure 8A). It has been observed that the composite electrode prepared has specific properties for quercetin, since compounds containing –OH and –NH_2_ groups, which will create an interference effect, do not generally work at this voltage value. It was determined that the fabricated ITO/MWCNT@PC@BiVO_4_ photosensitive sensor showed good selectivity in quercetin analysis.

**Figure 10:**
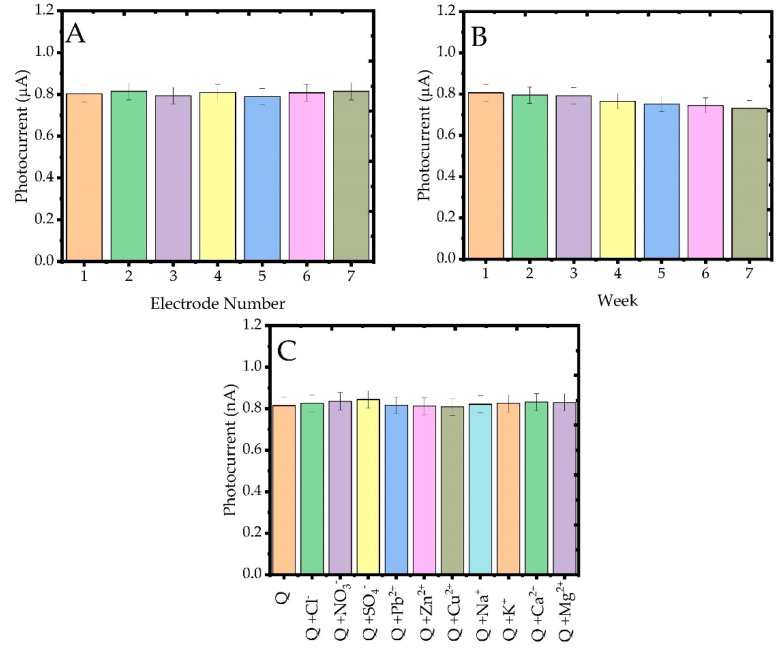
(**A**) Reproducibility, (**B**) long-term stability and (**C**) interference studies with different ions of the fabricated photosensitive quercetin sensors.

The reproducibility results of the prepared ITO/MWCNT@PC@BiVO_4_ composite electrode are shown in Figure 10A. It was calculated that seven different composite photosensitive sensors with the same properties and independently prepared under the same conditions had acceptable repeatability values and the RSD value was found to be 4.1%. Long-time stability measurement results are displayed in Figure 10B. It was seen that the photocurrent response of the ITO/MWCNT@PC@BiVO_4_ composite photosensitive sensor, which was stored and prepared at room conditions for seven weeks, preserved 92.4%. It is observed that the ITO/MWCNT@PC@BiVO_4_ photosensitive sensor prepared in this way has good selectivity, minimum interference effect and reproducibility in quercetin analysis. Some inorganic ions can coexist with quercetin in natural products, biological fluids and environmental samples and they may interfere with it during quercetin detection. The results of the study performed to investigate the interference influence of some inorganic ions (Cl^−^, NO_3_^−^, SO_4_^2−^, Pb^2+^, Zn^2+^, Cu^2+^, Na^+^, K^+^, Ca^2+^, Mg^2+^) on the quercetin analysis of the fabricated photosensitive sensor are shown in Figure 10C. In the interference study performed in the presence of the above-mentioned ions, it appears that transition metal ions such as Pb^2+^, Zn^2+^ and Cu^2+^ show almost no interference effect. On the other hand, it is observed that cations such as Na^+^, K^+^, Mg^2+^, Ca^2+^ and anions such as SO_4_^2−^, Cl^−^, NO_3_^−^, which are frequently found in food samples, have a very low level (0.1–0.8%) of interference effect. It was observed that the electrochemical response of the constructed ITO/MWCNT@PC@BiVO_4_ photosensitive sensor to quercetin was significantly preserved in the presence of these interference ions.

For quercetin analysis in a real-life sample, a sample of Turkish traditional black tea (ÇAY) was obtained from a local market. In our work, 1 g of black tea sample was treated with 50 mL of absolute ethanol and filtered through Whatman Grade 1 qualitative filter paper. Then, 1 mL of the obtained extract solution was taken and diluted with 25 mL of PBS and quercetin was analyzed by the developed photosensitive ITO/MWCNT@PC@BiVO_4_ sensor. In addition, the prepared ÇAY was analyzed by adding quercetin at various concentrations (0.1, 0.5, 1, 2, 5 µM) and RSD and recovery values were measured. The acquired quercetin analysis results in the black tea extracts via the developed photosensitive ITO/MWCNT@PC@BiVO_4_ sensor are given in Table 3. Additionally, prepared tea extracts were analyzed by U-HPLC (Agilent 1260 Infinity II). The equipment was included Phenomenex Kinetex 2.6 µm C18 100 Å HPLC columns, an automated gradient controller, a column oven and a diode array detector (DAD). The column oven temperature, injected sample volume and the flow rate was settled 30 °C, 20 µL and 1.5 mL/min. For HPLC measurements, the mobile phase was settled by isocratic elution; A (ultrapure water) 20% and acetonitrile (ACN) 80%. The mobile phase and samples were filtered by 0.45 mm pore size (Millipore) membrane filter. The standard quercetin at different concentrations was used to draw a calibration curve. Despite the added quercetin values as spikes, low RSD values and a good recovery were observed in the values taken. As seen in Table 3, the HPLC analysis results of the prepared black tea samples are quite close to the quercetin amounts estimated by the photosensitive sensors. This shows that the fabricated photosensitive ITO/MWCNT@PC@BiVO_4_ sensor works with good sensitivity for quercetin analysis in a real-life sample.

## 4. Conclusions

It is important to acquire quercetin, one of the antioxidants required for scavenging free radicals from the body, from natural sources. In the current study, a new photosensitive sensor was developed for the electrochemical analysis of quercetin from natural samples using ITO/MWCNT@PC@BiVO_4_ ternary composite electrode materials. Owing to the photosensitivity and stable structure of BiVO_4_ in the formed heterojunction electrode, the facilitation of electron conduction by PC, and the high electron permeability of MWCNT, electron transfer was successfully carried out in the developed photosensitive quercetin sensor. The photosensitive ITO/MWCNT@PC@BiVO_4_ quercetin sensor gave a linear response in the 10-200 µM quercetin concentration range at −0.8 V, and the LOD, LOQ and sensitivity values were correspondingly determined as 0.133 µM, 0.443 µM, 442 µA mM^−1^ cm^−2^. The developed photosensitive ITO/MWCNT@PC@BiVO_4_ sensor was found to have good selectivity, low interference effect, reproducible and high long-time stability values. In addition, this sensor in the real-life sample also has good recovery values. In summary, the developed photosensitive ITO/MWCNT@PC@BiVO_4_ sensor promises to perform a sensitive and stable quercetin analysis.

## Figures and Tables

**Table 1:** Electrochemical impedance characteristics of fabricated sensors.

Sensors	R_s_ (Ω)	R_CT_ (Ω)	C_μ_ (10^−7^ F cm^−2^)	f_max_ (Hz)	τ_e_ (ms)	τ_d_ (ms)
ITO	23.06	17.44	4.36	935.12	0.103	0.0760
ITO/MWCNT	18.02	104.84	1.05	268.02	0.594	0.1101
ITO/MWCNT@PC	22.05	66.13	1.69	223.03	0.713	0.1117
ITO/MWCNT@PC@BiVO_4_	16.01	60.75	2.02	79.12	2.012	0.1227

**Table 2:** Comparison of photosensitive sensors utilized in quercetin detection.

Sensors	Method	LOD (µM)	Linear Range (µM)	Ref.
ITO/MWCNT@PC@BiVO_4_	PEC	0.133	10-200	This study
BiVO_4_/FTO	PEC	0.154	100–10^6^	[58]
Nafion/MWCNT-GPE	PEC	6.0	10–910	[59]
TiO_2_/PtP/GCE	PEC	0.8	2–500	[21]
PPy/graphene	PEC	0.05	100–1000	[60]
*poly(Pyr-Carb)/ITO*	EC	0.59	2–500	[61]
Ac–Si/CPE	EC	0.0116	5–100	[62]
PtNPs/PEDOT-MeOH/GCE	EC	0.0052	0.04–99.09	[63]
NS-G/T-Ag@Au NPs/GCE	EC	0.05	0.5–15	[64]
CD/AuNPs/MWCNTs	EC	0.064	0.005–7	[65]

FTO: Fluorine doped tin oxide, GPE: Graphite pencil electrode, PtP: Platinum(II)-porphyrin complex, GCE: Glassy carbon electrode, PPy: Polypyrrole, Pyr-Carb: Poly(9-(2-(Pyren-1-Yl)Ethyl)-9H-Carbazole), CPE: Carbon paste electrode, PEDOT: Poly(3,4-ethylenedioxythiophene), NS-G/TAg: N, S Co-Doped Graphene/Ag@Au, CD: Cyclodextrin, PEC: photoelectrochemical, EC: electrochemical.

**Table 3:** Detection of quercetin in black tea extracts.

Extract	Added µM	Found µM	RSD %	Recovery %
Black Tea 1	0	0.61	3.6	-
	0.1	0.71	2.3	99.7
	0.5	1.11	2.6	99.8
	1	1.60	2.9	98.9
	2	2.56	3.6	97.8
	5	5.52	4.2	98.4
Black Tea 2	0	0.59	3.4	-
	0.1	0.68	2.6	99.6
	0.5	1.08	2.4	99.8
	1	1.58	2.2	98.8
	2	2.56	3.4	97.6
	5	5.55	5.2	96.5
Black Tea 1 HPLC	-	0.56	2.1	-
Black Tea 2 HPLC	-	0.53	1.8	-

## Data Availability

Not applicable.
